# Supplementary material for: Splice-Junction-Based Mapping of Alternative Isoforms in the Human Proteome
Source: Cell Rep. Author manuscript; Available in PMC 2020 Jan 15. (PMC6961840; doi:10.1016/j.celrep.2019.11.026)
Supplement: 3 [file NIHMS1546469-supplement-3.zip › DF2/PXD009737/TestisTrypsin-606-Q2KHT3-VDNQAMLHYIR.pdf]

A

Predicted sequence disorder and sequence features of Q2KHT3

Peptide: VDNQAMLHYIR Junction: sp|Q2KHT3|CL16A_HUMAN|ENSG00000038532|SE2|64383|chr16|10971230|10972559|+0|r54|T1 TrNovel: FALSE

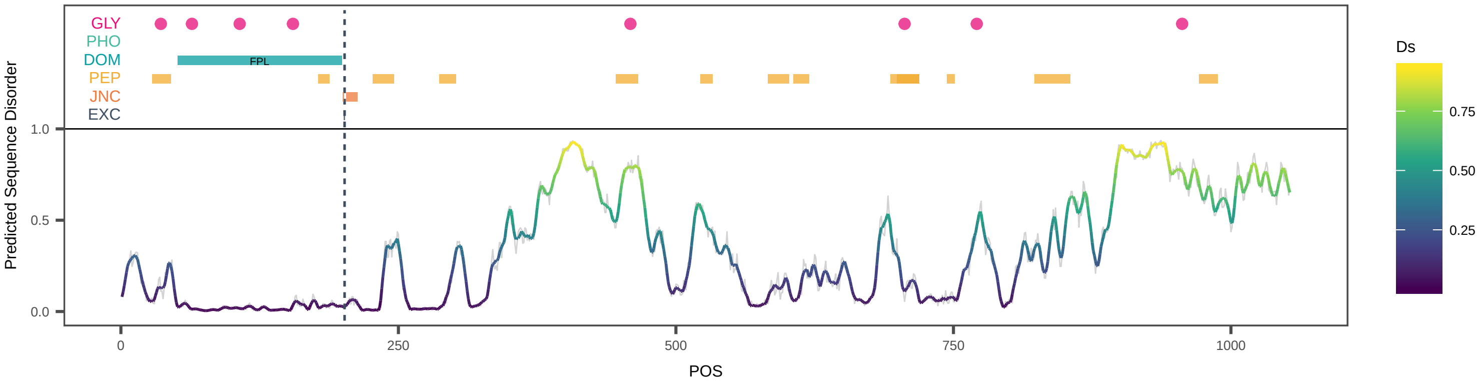

B

Distribution of sequence disorder in excised vs. mapped and non−excised regions of protein

M−W P−value vs. mapped: 0.036 vs. non−excised: 0.0874

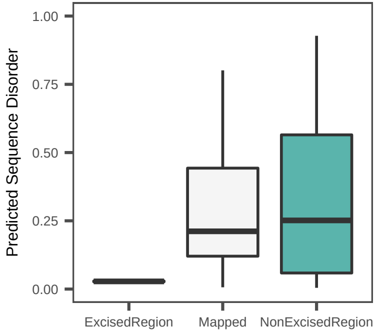

C
